# Effect of indirect ultrasonic activation on the root canal obturation with premixed calcium silicate cement: an in vitro study

**DOI:** 10.1186/s12903-025-05748-2

**Published:** 2025-04-15

**Authors:** Bokyung Shin, Won-Jun Shon, Yeon-Jee Yoo

**Affiliations:** 1 Department of Conservative Dentistry, Section of Dentistry, Seoul National University Bundang Hospital, Seongnam, Republic of Korea; 2 Department of Conservative Dentistry, National Health Insurance Service Ilsan Hospital, Goyang, Republic of Korea; 3 Department of Conservative Dentistry, Dental Research Institute, Seoul National University School of Dentistry, Daehak-ro 101, Jongro-gu, Seoul, Republic of Korea

**Keywords:** Indirect ultrasonic activation, Leakage, Premixed calcium silicate cement, Root Canal obturation, Void

## Abstract

**Background:**

To assess the effect of indirect ultrasonic activation on the root canal obturation with a premixed calcium silicate cement (CSC).

**Methods:**

Twenty-six single-rooted premolars were sectioned to the length of 11 mm. All the roots were instrumented using ProTaper Next instruments and randomly assigned to either the control (*n* = 4) or experimental (*n* = 22) groups according to root canal obturation methods. Root specimens allocated to experimental groups were obturated with Endocem MTA, with (group EMU) or without (group EM) indirect ultrasonic activation (*n* = 11/group). Root specimens obturated with gutta-percha served as positive and negative (after nail varnish coating) controls. Intraoral radiograph images were used to assess the presence or absence of voids in the coronal, middle, and apical thirds of each specimen. The specimens were connected to a nanoscale fluid filtration device to measure quantitative leakage data. Data was statistically analyzed with a significance level of 0.05.

**Results:**

There was no significant difference in the voids formation between the two groups (*p* > 0.05), but specimens in group EMU presented significantly higher quantitative leakage than those in group EM (*p* < 0.05).

**Conclusions:**

Within the limitation of this study, indirect ultrasonic activation of premixed CSC did not affect the void formation but presented higher quantitative leakage. Future research incorporating micro-CT imaging would enable a more precise and comprehensive analysis, providing valuable insights into the root canal obturation with premixed CSCs.

## Introduction

Apical periodontitis refers to an inflammatory response of the periradicular tissues triggered by bacterial infection within the root canal. The presence of preoperative apical periodontitis is considered an adverse indicator of the success of root canal treatment, often leading to endodontic failure. A meta-analysis assessing the success of endodontic procedures revealed that certain factors significantly affect the outcome of primary root canal treatment. These include the absence of periapical radiolucency preoperatively, a well-filled root canal without voids, length of the root filling within 2 mm from the radiographic apex, and satisfactory coronal restoration [1].

A systematic review focusing on mineral trioxide aggregate (MTA) highlighted its effectiveness as a root canal filling material in both primary and permanent teeth [2]. However, orthograde MTA filling resulted in lower quality than conventional techniques using gutta-percha and AH Plus sealers, as indicated by an in vitro study [3]. Additionally, a meta-analysis underscored the significant influence of voids in root canal fillings on the success rate of endodontic treatment [1]. Consequently, endeavors are underway to increase the quality of root canal filling with MTA.

Previous research has demonstrated that additional ultrasonic activation during orthograde MTA filling leads to a reduction in void volume compared with hand condensation alone [4, 5]. Furthermore, bacterial leakage was lower in the ultrasonically placed MTA group than in the nonultrasonic MTA group [6]. According to recent systematic review on ultrasonic activation of endodontic sealer, bond strength was higher and more variable in the ultrasonically activated group [7]. Also, most studies showed that ultrasonic method increased intratubular penetration in the root canal [7].

In a prior study, Yeung et al. reported that hand condensation of MTA with indirect ultrasonic activation resulted in a statistically significant increase in the MTA fill density compared with hand condensation without indirect ultrasonic activation [8]. The study recommended applying indirect ultrasonic activation for 1 second at the lowest power setting using an ultrasonic unit. Additionally, Keles et al. demonstrated that while the root filling depth was similar at the apical level with or without the indirect ultrasonic activation, denser filling was observed in the coronal isthmus with the indirect ultrasonic activation via micro-CT evaluations [5]. However, conflicting results have been reported in other studies, which noted more voids and gaps in the indirect ultrasonic activation group [9, 10].

Calcium silicate-based cements (CSCs), featuring pure tricalcium silicate, have emerged as promising alternatives to traditional endodontic filling materials, addressing the limitations associated with the MTA [2, 11–13]. These bioceramics primarily comprise synthetic tricalcium silicate and are devoid of aluminum, resulting in heightened purity and biological activity compared with those of Portland cement-based materials [11, 14, 15]. The introduction of premixed CSC into the market offers increased convenience to users, as it can be utilized directly without the need for mixing. This type of CSC provides several advantages, including user-friendliness and a consistent mixing ratio of materials. In systematic review [16], premixed calcium silicate-based sealers have exhibited favorable physicochemical and biological properties in vitro. Overall, their performance has been found to be comparable to or even superior to that of traditional endodontic sealers, as evidenced by studies conducted in both in vitro and in vivo animal models [16].

This study aimed to investigate the effect of ultrasound on the obturation quality of premixed CSC in root canals. Indirect ultrasonic activation was applied on premixed CSC during root canal filling, and the sealing ability of the root canal filling was observed via a real-time nanoleakage device. The first null hypothesis was that the application of ultrasound during root canal obturation with premixed CSC does not affect the quality of the root canal filling. The second hypothesis was that ultrasound application during root canal obturation with premixed CSC does not affect microleakage within the root canal.

## Methods

### Ethics approval

The present study was approved by the Ethics Committee and Institutional Review Board (IRB) of the National Health Insurance Service Ilsan Hospital, Korea (IRB No. 2022-03-042). Informed written consent was obtained from all participants, and the extractions were preplanned with their consent.

### Sample size calculation

A sample size calculation was performed with G*Power 3.1.9 (Universität Kiel, Kiel, Germany) to detect significant differences between groups. The estimated sample size was 8 teeth per group (effect size: 1.310, alpha: 0.05, 80% power). A total of 26 teeth were prepared for the experiment, with 11 teeth in each group, including 2 for the positive control and 2 for the negative control.

### Sample Preparation

A total of 26 human permanent premolars were obtained with consent, which were preplanned with consent. Patients older than 19 years were included. The extracted teeth were stored in saline at 37 °C for up to 2 months. Root inspection was carried out via an OMPI Pico endodontic optical microscope (Carl Zeiss Meditec Inc., Jena, Germany) at a magnification of 12.5× to exclude teeth with cracks, defects, severe canal calcifications, or a history of prior endodontic treatment.

**Fig. 1:**
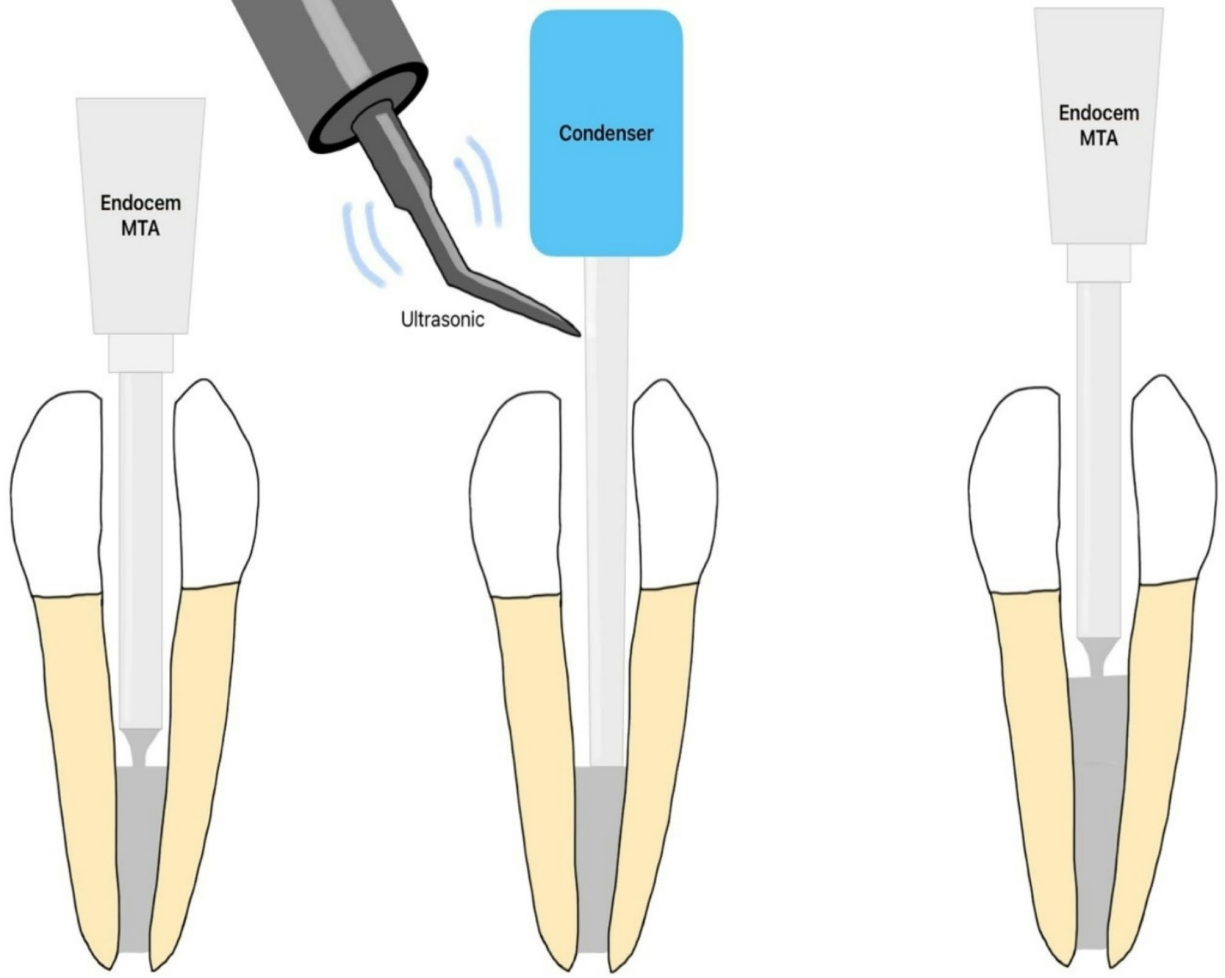
The procedure of root canal filling via indirect ultrasonic activation

After decoronation, the roots were sectioned to a length of 11 mm and affixed to a water-saturated porous sponge to replicate a periapical environment. Apical patency was confirmed via a #10 stainless steel K-file (Dentsply Sirona, Charlotte, NC, USA), and the working length was set to 1.0 mm short of the apical foramen. Root canal instrumentation was performed using ProTaper Next X1, X2, and X3 instruments (Dentsply Sirona), with copious irrigation of the root canals using 3.5% sodium hypochlorite (NaOCl) delivered through a 27-gauge side-cutting irrigator tip (Ultradent, South Jordan, UT, USA). The final irrigation consisted of 5 mL of 17% ethylenediaminetetraacetic acid (EDTA) followed by 5 mL of 3.5% NaOCl. The root canals were then dried using paper points (Diadent, Almere, Netherlands). Prepared root specimens were randomly assigned to either the experimental (*n* = 22) or control (*n* = 4) groups according to root canal obturation methods. Root specimens allocated to experimental groups were obturated with Endocem MTA (≈ 7.5 mm^3^/specimen), with (group EMU) or without (group EM) indirect ultrasonic activation (*n* = 11/group). In group EMU, root canals were obturated by Endocem MTA (Maruchi, Wonju, Korea) with the indirect ultrasonic activation (ProUltra Endo-1 ultrasonic tip; Dentsply Sirona) via an endodontic plugger (BL-S Kondenser 40/80; B&L Biotech, Ansan, Korea) and an ultrasound unit (P5 XS Newtron; Satelec Acteon, Mérignac, France) at the lowest power setting for 1 second following the Yeung’s study [8] (Fig. 1). In the EM group (*n* = 11), the root canals were obturated by Endocem MTA without indirect ultrasonic activation. Teeth allocated in control groups (*n* = 2/group) were obturated with gutta-percha (Dentsply Maillefer, Ballaigues, Switzerland) using the continuous wave condensation technique without the use of sealer. For negative control group, the specimens were completely coated with two layers of nail polish, including the apical foramina, to prevent any fluid movement through the specimens.

### Radiographic assessment

The quality of root canal filling was assessed based on the presence or absence of voids using 2-dimensional periapical radiograph. Each specimen was examined on a diagnostic screen for the presence of voids in the coronal, middle, and apical thirds. The observer was blinded to the root canal filling technique.

### Leakage test

**Fig. 2:**
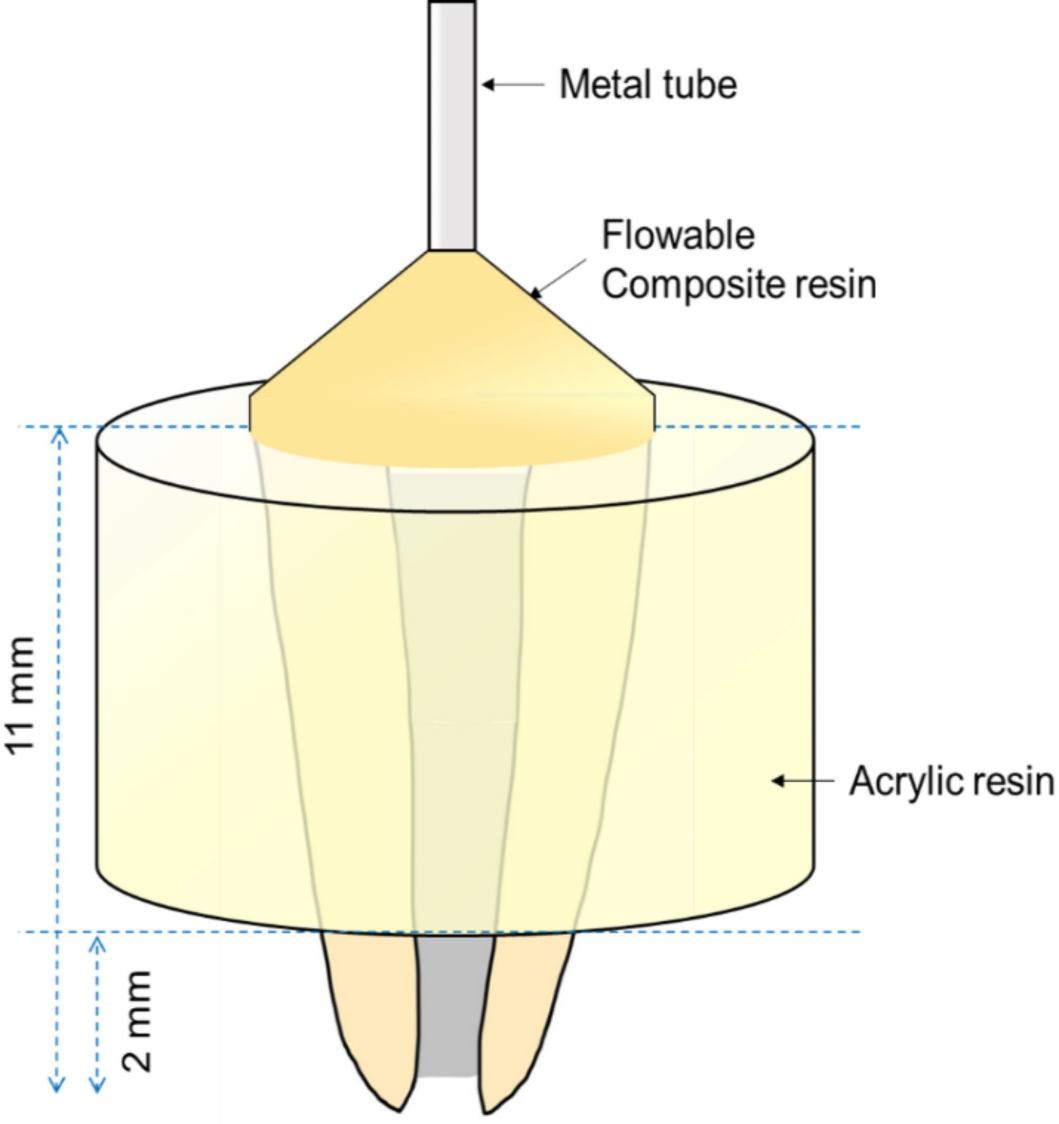
Specimen preparation for the leakage test

**Fig. 3:**
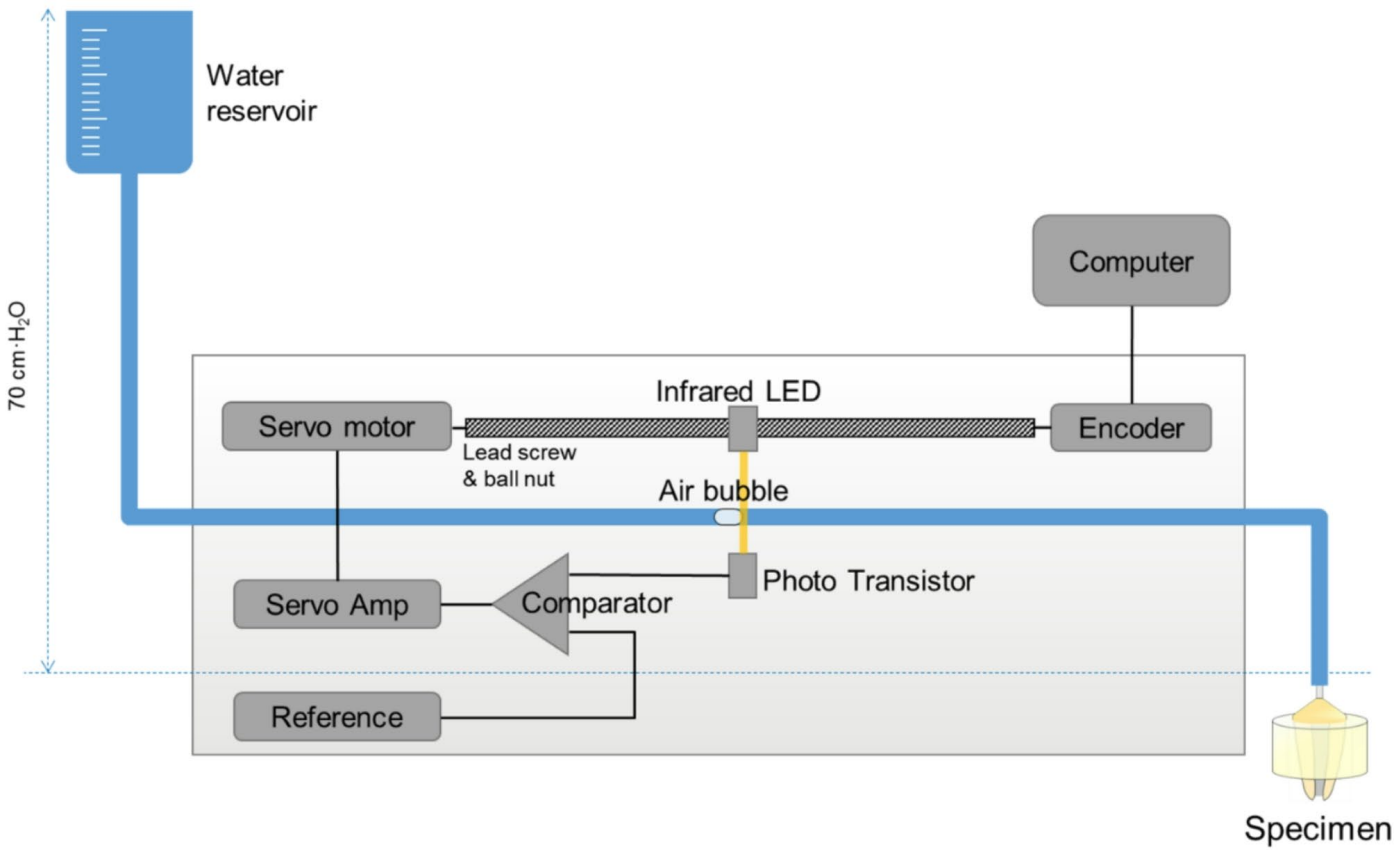
Schematic diagram of the nanoscale fluid filtration device nanoflow

The quantitative apical leakage of each specimen was measured after one week of root canal obturation via Nanoflow (IB System, Seoul, Korea), a nanoscale fluid filtration device, according to previously reported [17]. In summary, the coronal part of the root was affixed to a sandblasted metal tube via flowable composite resin, and the filled roots were embedded into acrylic resin, with the exception of the most apical 2 mm, to prevent undesirable leakage (Fig. 2). A hydrostatic pressure of 70 cm∙H_2_O was applied throughout the measurement process with a water reservoir. Finally, the prepared sample was connected to the measuring device. The nanoflow device recorded the fluid flow for up to 30 min at 0.5-second intervals via a PCI 6016 data acquisition device (National Instrument, Austin, TX, USA) (Fig. 3) [18].

### Statistical analysis

Comparisons between groups for quantitative leakage and presence of voids were performed using Student’s t-test and Chi-square test with a significance level of 5%. The statistical analysis was performed via SPSS ver. 25 (SPSS Inc., Chicago, IL, USA).

## Results

### Radiographic assessment

No technique used for compaction of premixed CSCs could consistently produce void-free root canal obturations. Table 1 shows the percentage of specimens with void(s) (%) in radiographic assessment. A total of 6 out of 11 specimens in group EMU and 7 out of 11 specimens in group EM were free of voids in radiographic assessment (*p* > 0.05). Within each group, presence of voids were significantly different according to the level from apex (*p* < 0.1; group EM, 4.714; group EMU, χ^2^ = 5.802). Within each level from the apex, specimens without indirect ultrasonic activation presented significantly higher incidence of voids in apical third (*p* < 0.1; χ^2^ = 3.667).

**Table 1:** Radiographic assessment of root Canal obturation

		Presence of void (%)	
		Group EMU	Group EM
Overall		54.5^A^	63.6^A^
Level from the apex	Apical	9.1^bβ^	45.5^aα^
	Middle	45.5^aα^	63.6^aα^
	Coronal	9.1^aβ^	18.2^aβ^

Same uppercase superscript indicates significant difference between the two groups (*p* < 0.05)

Same lowercase superscript indicates significant difference between the two groups at the same level from the apex (*p* < 0.1)

Same Greek superscript indicates significant difference between the level from the apex within the group (*p* < 0.1)

### Leakage test

**Fig. 4:**
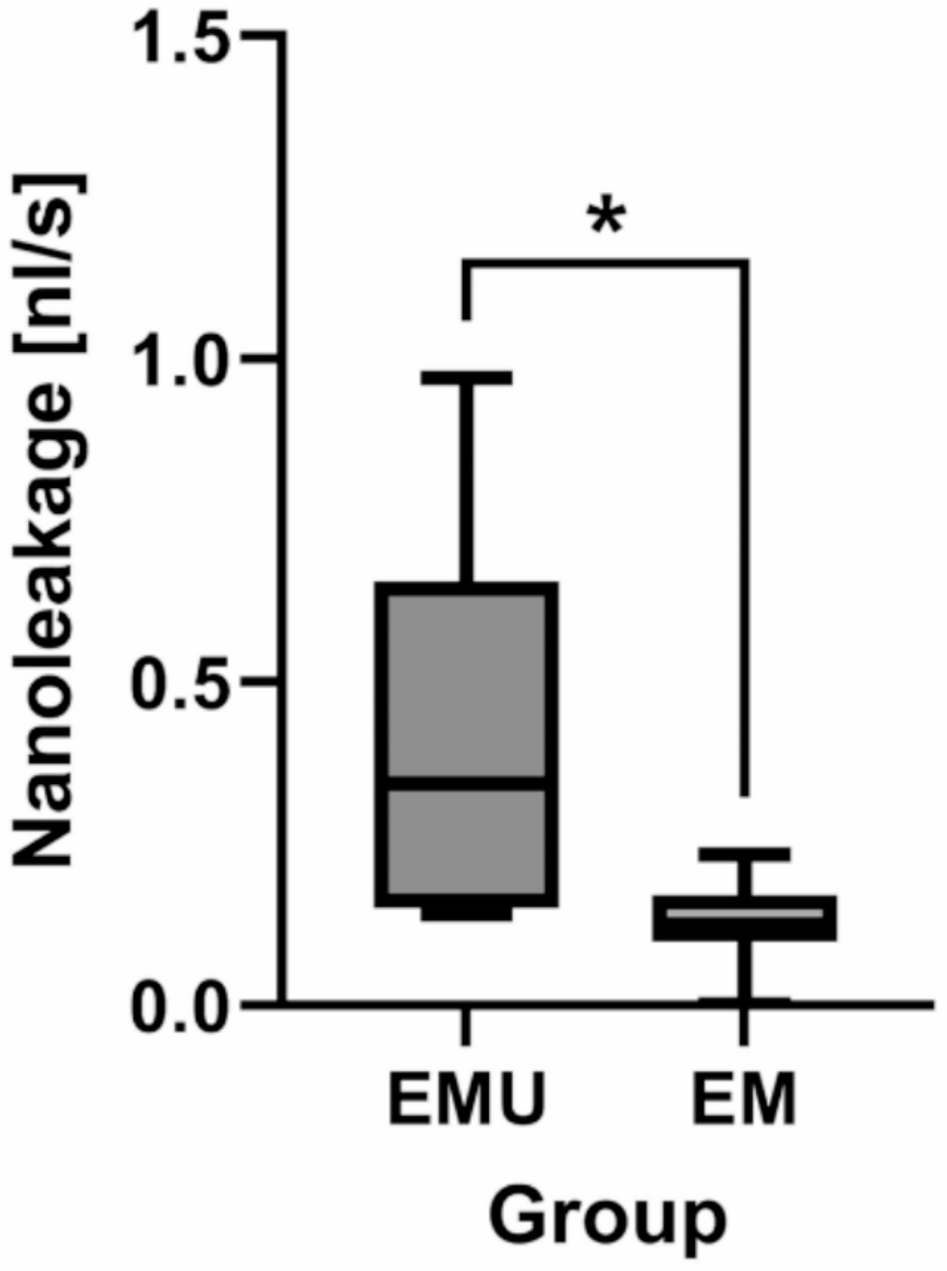
Quantitative nanoleakage of premixed CSCs in root canals. Asterisk (*) indicates statistical difference (*p* < 0.05)

The apical microleakage values of the control groups validated the reliability of the experimental fluid filtration model. No fluid movement was detected in the negative controls, confirming the absence of leakage within the device, while the positive controls (without sealer) exhibited rapid and immeasurable fluid movement, representing complete (100%) leakage. There was a significant difference between the groups EMU and EM (*p* < 0.05) [95% CI: 0.08–0.47] [effect size (Cohen’s d): 1.33, 95% CI: 0.34–2.31] (Fig. 4).

## Discussion

This study investigated the use of ultrasound during root canal filling with premixed CSCs to evaluate its effect on obturation quality and microleakage within the root canal. The application of ultrasound did not improve the quality of obturation but led to a reduction in sealing ability against microleakage. As a result, the first null hypothesis was accepted while the second null hypothesis was rejected. These findings suggest that the use of ultrasound during root canal filling with this material may require further investigation to better understand its limitations and identify optimal conditions for its application.

The main goal of root canal filling is to achieve a hermetic seal in order to prevent microbial contamination [19]. Systematic review on root canal filling has reported the correlation of voids in the root canal and success rate of endodontic treatment [20]. Thus, it is important to completely seal the root canal system and minimize the void or gap formation during root canal filling. However, completely filling the root canal with MTA is very challenging, and void or gap is commonly found in orthograde MTA filling compared to conventional root canal filling using gutta percha and root canal sealer [4, 21]. In order to enhance the quality of MTA orthograde fillings, several methods have been attempted, such as, hand condensation, reverse rotary motion of NiTi files, and ultrasound activation [4, 21].

In previous studies, ultrasound was applied to MTA mixed as a powder-liquid mixture, in order to enhance the quality of root canal filling [8, 9]. Yeung et al.. attempted to densely pack MTA by applying ultrasound with minimal power to the MTA after hand condensation using a plugger for one second [8]. Such attempts have also been made in studies by Keles and El-Ma’aita, who reported successful outcomes; however, conversely, more voids and gaps have been observed [5, 9, 10]. In this study, ultrasound activation was applied to premixed CSCs to examine its effects during root canal filling. The procedure used the same 1-second lowest ultrasonic power, indirect ultrasound, and manually compaction method, based on Yeung’s method from a previous study.

This study aimed to evaluate the effects of ultrasound activation specifically when using premixed CSCs for root canal filling. The effectiveness of ultrasonic activation in powder-liquid mixed MTA remains controversial [8–10]. On the other hand, the use of ultrasonic activation has been reported to be effective in several studies when GP and sealer combinations are used for root canal filling. For example, in C-shaped teeth, indirect sealer activation with ultrasound has been shown to be effective [22]. Additionally, in cases with two root canals, the use of ultrasonic activation with the single-cone technique has been found to improve the quality of the root canal filling [23]. A systematic review on bond strength and intratubular penetration also reported that both bond strength and intratubular penetration improved with the application of ultrasound [7].

New CSCs are continuously being developed and introduced to the market. The original MTA formulation consisted of radiopacified Portland cement, whereas CSCs are consisted of pure tricalcium silicate [24]. To enhance the biointeractive and biological properties of CSCs, the proportion of bioactive calcium silicate particles has increased, and the particle size has decreased [14, 25]. Additionally, to improve physical characteristics, aluminum has been eliminated, alternative radiopacifiers with additives have been included, and the particle size has decreased [24]. In the original MTA formulations, bismuth oxide was used as a radiopacifier, resulting in significant discoloration and periapical tissue toxicity. In contrast, CSCs utilize zirconium dioxide and other alternatives as radiopacifiers to address staining and toxicity concerns [24]. Recently, the premixed type of CSC has been introduced with several advantages, especially in terms of convenience [24]. According to a systematic review, premixed CSC exhibit favorable physicochemical and biological properties in vitro. Generally, their performance is comparable to or even superior to that of traditional endodontic sealers, as demonstrated in both in vitro and in vivo animal studies [16].

This is the first study to evaluate the effect of ultrasonic activation during root canal filling on the leakage of premixed CSCs. The experiment initially anticipated a reduction in leakage with the application of ultrasound, but this expectation was not met, as the leakage of indirect ultrasonic activation samples showed significantly higher leakage than the samples without indirect ultrasonic activation. Not only the sealing ability decreased, but also the leakage increased with more variance.

The presence of voids in the specimens was evaluated via two-dimensional imaging and the results showed similar incidence of voids between the two groups. Regarding the level from the apex, indirect ultrasonic activation of premixed CSCs showed significantly higher incidence of voids-free specimens (*p* < 0.05). In the middle third, half of the specimens from both groups showed voids, which appear to have occurred during the process of adding the premixed CSCs after the obturation of the apical third. Further research is necessary to determine the optimal clinical applications of ultrasound activation in root canal obturation.

The results suggest a need to reconsider the clinical utility of the leakage test. Despite the previously noted shortcomings of leakage tests [26, 27], recent publications have continued to introduce studies utilizing the fluid filtration method [28, 29]. The Nanoflow device utilized in this experiment is based on the previous fluid filtration method known as the Flodec method, developed by Pashley [30], and recent studies reported that it could measure a real time leakage with a precision of 0.196 nL resolution [28, 29]. In this study, real-time measurement under hydrostatic pressure using the device allowed precise tracking of fluid flow through obturated root canals, revealing a significant difference when the premixed CSC was condensed with indirect ultrasonic activation. However, the clinical relevance of the results remains to be investigated further in a proof-of-concept study [31]. The current study has several limitations. First, the methodology of this study lacks micro-CT assessments and the evaluation of root canal obturation was based on two-dimensional x-rays and quantitative leakage measurements with fluid filtration device. Recent studies have utilized micro-CT to assess the quality of filling in a more accurate and quantitative three-dimensional manner [32, 33]. The absence of micro-CT analysis in this study may limit the comprehensive assessment of void distribution and material adaptation. Therefore, future evaluations using micro-CT may be necessary. Additionally, calcium silicate materials are sensitive to moisture, and recent research has suggested the use of specific drying protocols [34, 35]. However, in this study, the root canals were completely dried before filling, which could have adversely affected the material’s setting and, consequently, sealing ability. Moreover, anatomical variations in teeth could have influenced the results [31]. While this is a common issue in all in vitro studies, it would have been beneficial to select teeth on the basis of accurate anatomical assessments via micro-CT scans before experimentation. However, difficulties in obtaining extracted teeth for the study made it challenging to consider tooth morphology as a criterion. An editorial of the International Endodontic Journal has discussed the importance of tooth morphology for root filling and leakage studies [31]. De-Deus advised the use of paired teeth and high-resolution x-ray tomography to improve the accuracy of the test. Therefore, for future experiments, efforts to collect teeth with similar morphologies and pair the teeth may be necessary.

## Conclusions

Indirect ultrasonic activation of premixed CSC during root canal obturation did not significantly affect the void formation but was associated with significantly higher quantitative leakage. Taken together, further research is needed to assess the clinical relevance of indirect ultrasonic activation of premixed CSCs in root canal obturation, particularly regarding its reliability and its long-term clinical outcomes.

## Data Availability

The data used and analyzed during the current study are available from the corresponding author upon reasonable request.
